# Prediction of sentinel lymph node status in patients with early breast cancer using breast imaging as an alternative to surgical staging—a systematic review and meta-analysis

**DOI:** 10.1186/s13643-025-03005-9

**Published:** 2025-11-25

**Authors:** Cornelia Rejmer, Malin Hjärtström, Pär-Ola Bendahl, Looket Dihge, Ida Skarping, Daqu Zhang, Magnus Dustler, Lisa Rydén

**Affiliations:** 1 Department of Clinical Sciences, Division of Surgery, Lund University, Lund, Sweden; 2 Department of Clinical Sciences Lund, Anesthesiology and Intensive Care, Lund University, Lund, Sweden; 3 Department of Clinical Sciences Lund, Division of Oncology, Lund University, Lund, Sweden; 4 Department of Plastic and Reconstructive Surgery, Skåne University Hospital, Malmö, Sweden; 5 Department of Clinical Physiology and Nuclear Medicine, Skåne University Hospital, Lund, Sweden; 6 Centre for Environmental and Climate Science, Computational Science for Health and Environment, Lund University, Lund, Sweden; 7 Department of Translational Medicine, Diagnostic Radiology, Lund University, Lund, Malmö, Sweden; 8 Department of Surgery and Gastroenterology, Skåne University Hospital, Malmö, Sweden

**Keywords:** Breast cancer, Prediction model, Breast imaging, Lymphatic metastasis, Systematic review, Meta-analysis

## Abstract

**Background:**

Prediction models for sentinel lymph node (SLN) status could potentially substitute surgical axillary staging in patients with early breast cancer. Several imaging modalities have been used with various feature extraction and selection approaches. This systematic review and meta-analysis aimed to evaluate prediction models for SLN status based on breast imaging in patients with early breast cancer to summarize the current evidence and to identify areas requiring additional research.

**Methods:**

The systematic literature search strategy was based on the Population, Intervention, Comparison, and Outcome (PICO) framework: P: female patients with clinically node-negative invasive breast cancer scheduled to undergo primary surgery; I: breast imaging; C: upfront sentinel lymph node biopsy; and O: prediction model performance regarding SLN status. The search was conducted in the PubMed, Embase, Web of Science, Cochrane, and Cumulative Index to Nursing and Allied Health Literature databases in March 2024. The screening of records, data collection, and bias assessments were performed independently by two reviewers. The risk of bias was assessed via the Quality Assessment of Diagnostic Accuracy Studies 2 (QUADAS-2) tool and the Prediction Model Study Risk of Bias Assessment Tool. A meta-analysis was performed using the random-effects model to assess performance and heterogeneity overall and in subgroups.

**Results:**

The literature search resulted in the inclusion of 32 articles with 11,464 patients in total. Five imaging categories were included: ultrasound (*n* = 8), magnetic resonance imaging (MRI) (*n* = 17), mammography (*n* = 1), positron emission tomography computed tomography (*n* = 1), and multiple modalities (*n* = 5). Four studies, assessed as having a high risk of bias, were excluded from the meta-analysis. The meta-analysis revealed heterogeneity in overall performance, except for MRI-based studies, with a pooled area under the curve of 0.85 (95% confidence interval 0.82–0.87). Meta-regression indicated that MRI and model calibration assessment upon validation contributed to heterogeneity.

**Conclusions:**

This systematic review and meta-analysis revealed that prediction models using breast imaging—particularly MRI—could serve as a noninvasive alternative to surgical axillary staging in patients with early breast cancer. The results illustrate the heterogeneity between studies and the need for additional high-quality studies.

**Systematic review registration:**

PROSPERO CRD42022301852, available at https://www.crd.york.ac.uk/PROSPERO

**Supplementary Information:**

The online version contains supplementary material available at 10.1186/s13643-025-03005-9.

## Background

Sentinel lymph node biopsy (SLNB) is currently the standard of care for surgical axillary staging and is recommended for patients with clinically node-negative (cN0) early breast cancer [1–4]. Although SLNB is commonly performed in all women with cN0 breast cancer, only 15–25% have sentinel lymph node metastasis [5–7], meaning that the majority do not receive any therapeutic effect from the intervention. Hence, the omission of SLNB has, in recent years, been increasingly debated for specific patient groups [4]. Furthermore, the randomized controlled trials on the omission of SLNB in patients with early breast cancer, including the Sentinel Node vs. Observation After Axillary Ultra-Sound (SOUND) trial [6] and the Intergroup Sentinel Mamma (INSEMA) study [8], have shown that the omission of SLNB leads to noninferior distant disease-free and invasive disease-free survival, respectively, at 5 years of follow-up. Additionally, the procedure is associated with a risk of complications such as lymphedema, numbness and reduced arm mobility [9]. The omission of SLNB reduces complication rates and increases quality of life, as shown by Reimer et al. [5].

In recent years, noninvasive methods for axillary staging such as prediction models using clinicopathological data and imaging data have been developed. However, studies have demonstrated extensive heterogeneity regarding the selection of patients, the choice of imaging modality, the methods of feature extraction from images, and the statistical modeling approach, thus making direct comparisons challenging [10–16]. Commonly used imaging modalities in clinical work-up, including digital mammography (DM) and ultrasound (US), as well as more advanced imaging modalities, such as magnetic resonance imaging (MRI), computed tomography (CT), digital breast tomosynthesis (DBT), and positron emission tomography (PET), have been evaluated for axillary staging [2, 4, 17]. The methods used for feature extraction vary from manual extraction by a radiologist to fully automated approaches using machine learning algorithms. Additionally, the statistical methods for model development vary across studies, ranging from logistic regression and penalized logistic regression using the least absolute shrinkage and selection operator (LASSO) to more advanced methods such as deep learning [13–16]. Previous reviews and meta-analyses on the use of imaging data in prediction models for axillary lymph node metastasis have focused on a broad population of patients with breast cancer, including both early and locally advanced breast cancer, or on a specific imaging modality [17–20]. A study by Gong et al. [17] published in 2022 evaluated radiomics models for the prediction of lymph node metastasis in patients with breast cancer. Although the study evaluated several imaging modalities, the patient population included all types and stages of breast cancer, all types of surgical axillary staging, and both men and women, thus reducing the clinical relevance and interpretability of the results. To the best of the authors’ knowledge, no systematic review has specifically synthesized breast imaging-based prediction models for sentinel lymph node (SLN) status in patients with early-stage female breast cancer.

While axillary ultrasound (AUS) is currently recommended as part of the initial clinical work-up in some countries, it is not recommended in others. Consequently, the definition of cN0 breast cancer varies, as some studies include AUS findings, whereas others do not [1, 2, 4]. In contrast, breast imaging is an integral part of the standard diagnostic work-up for breast cancer and is implemented worldwide. Given its universal accessibility and routine use, breast imaging may provide a more standardized and broadly applicable approach for predicting SLN status than AUS.

Therefore, the current systematic review and meta-analysis aimed to evaluate breast imaging-based prediction models for sentinel lymph node (SLN) status in women with early-stage breast cancer scheduled for primary surgery, synthesize current evidence and to identify key areas for future clinical research.

## Materials and methods

A protocol for the systematic review was registered at the International Prospective Register of Systematic Reviews (PROSPERO) (CRD42022301852) prior to study initiation. It was subsequently revised following PROSPERO reviewer feedback and continuously updated throughout the review process. This study was conducted in accordance with the Preferred Reporting Items for Systematic Reviews and Meta-analysis (PRISMA) checklist for systematic reviews [21, 22] (Supplementary 1) and the guidelines for conducting a systematic review and meta-analysis on prediction model performance [23]. Studies were selected on the basis of the following framework: participants, index test, comparator, outcomes, and type of study. Eligibility criteria were established to identify women with early breast cancer who were scheduled to undergo SLNB in accordance with clinical guidelines [1, 4], as previously described.

### Eligibility criteria

Inclusion criteria:

1. Original articles published in English
2. Female patients with cN0 invasive breast cancer, who were scheduled to undergo primary surgery
3. Breast imaging (with or without imaging of the axilla) performed before primary surgery: mammography, ultrasound, magnetic resonance imaging, computed tomography, and/or other
4. SLNB only or SLNB followed by axillary lymph node dissection (ALND)

Exclusion criteria:

1. Meta-analyses, systematic reviews, reviews, editorials, or commentaries
2. Patients receiving neoadjuvant therapy, with clinically node-positive breast cancer, male sex, cancer in situ, distant metastasis, or recurrent locoregional breast cancer
3. Only imaging of the axilla or imaging of the breast without feature extraction
4. ALND without SLNB or no data on SLN status

For studies in which information about clinical nodal status was missing or unclear, participants who underwent upfront surgery and SLNB were assumed to have cN0 breast cancer. Similarly, in studies that did not explicitly state the method for determining SLN status (SLNB or ALND), but clearly mentioned investigating SLNB, the patients were assumed to have undergone SLNB. If necessary, the corresponding author of the article was contacted by one of the reviewers (CR, MH, or MD) to obtain additional information.

### Literature search and study selection

A systematic literature search was conducted by the Lund University Library from December 2021 to January 2022 and was updated in March 2024 to ensure comprehensive coverage. The search strategy (Supplementary 2) was filtered to include females only. The following databases were searched: PubMed, Embase, Web of Science, the Cochrane Library, and the Cumulative Index to Nursing and Allied Health Literature (CINAHL). All retrieved records were screened for eligibility by two independent reviewers (CR and either MD or MH) based on the predefined eligibility criteria using Covidence systematic review software (2024, Veritas Health Innovation, Melbourne, Australia). Disagreements between reviewers were resolved via discussion. If a consensus could not be reached, a third reviewer (LR) was consulted. To identify gray literature, additional searches were conducted by two independent reviewers (CR and MH) from November to December 2022 in the Clinical Trials, Digitala vetenskapliga arkivet (DIVA), Libris, and Grey Matters databases. The reference lists and citations of all included articles were manually screened by two independent reviewers I(CR and either MD or MH) to identify additional eligible articles meeting the inclusion criteria.

### Data extraction

Data extraction was performed via the Covidence systematic review software. The data were extracted independently by two reviewers (CR and MD) using an extraction form based on the CHecklist for critical Appraisal and data extraction for systematic Review of prediction Modeling Studies (CHARMS) [24]. Disagreements were resolved through discussion until a consensus was reached.

The primary outcome was the area under the curve (AUC) for predicting SLN status; for a binary outcome, the AUC is equivalent to the C-index. Additional performance metrics included sensitivity, specificity, true positives, true negatives, false positives, false negatives, and the false-negative rate.

### Risk of bias assessment

The risk of bias was assessed via the Quality Assessment of Diagnostic Accuracy Studies 2 (QUADAS-2) tool [25], per the protocol and the Cochrane recommendations at the time of protocol registration. The Prediction Model Study Risk of Bias Assessment Tool (PROBAST) [26] was used to specifically assess the risk of bias in studies on prediction models, despite not being mentioned in the study protocol. Bias assessments were performed independently by two reviewers (CR and MD), and all disagreements were resolved by discussion until a consensus was reached. Studies identified as having a high risk of bias in at least one domain of the QUADAS-2 were excluded from the meta-analysis. However, studies assessed as having a high risk of bias via PROBAST were not excluded, in accordance with the protocol.

### Subgroups

Subgroup analyses were performed on the basis of imaging modality (US, MRI, or other/multiple modalities) and the included types of data (imaging only or clinical + imaging).

### Meta-analysis

Bias assessments were illustrated by a traffic light plot and a risk of bias graph. The two tools for assessing the risk of bias were compared via an exact test of symmetry. For this purpose, the bias assessments were handled as ordinal variables. Approximate standard deviations and 95% confidence intervals (CIs) for the AUCs were calculated for all studies included in the meta-analysis [23] for comparability as CIs were missing in several of the publications. CIs were calculated on the logit scale to ensure that the upper CI limits were ≤ 1.0. The logit scale was also used in the meta-analysis. For studies providing AUCs for both training and validation, the validation (external if available) AUC was selected for analysis. Pooled AUCs were calculated using a random effects model and illustrated by forest plots for all studies and were stratified by subgroup. Heterogeneity between studies was assessed with I^2^, Tau^2^, and Cochran’s *Q*. Univariable meta-regression analysis using a random effects model was used to evaluate potential causes of heterogeneity. The following variables were evaluated: country and year of publication, imaging modality (US, MRI, or other/multiple modalities), whether imaging of the axilla was performed or not, whether single or multiple imaging modalities were used, the type of data included (imaging only versus clinical + imaging), the region of interest (ROI) preprocessing approach (manual, semiautomated, or automated using artificial intelligence (AI)), the method of feature extraction (manual, semiautomated, or automated using AI), the method of model development (deep learning versus other), validation of the prediction model (none, internal only, or external), model calibration assessment upon validation (yes versus no), the total study sample size, the proportion of pathologically node-positive (pN +) patients, and the risk of bias assessment (QUADAS-2 tool and PROBAST), categorized as low, unclear, or high risk. Additionally, a multivariable meta-regression on imaging modality was performed, adjusting for the type of validation, the proportion of pN + patients, and the total study sample size. Bubble plots illustrating the associations between the AUC and key covariates were developed. The sizes of the bubbles are inversely proportional to the variances of the AUC estimates. A sensitivity analysis regarding overall performance and heterogeneity was conducted to estimate the effect of excluding studies assessed as having a high risk of bias. Publication bias was visualized via a funnel plot and Egger’s test. Statistical analyses were performed using Review Manager (The Cochrane Collaboration, 2024. *Review Manager* (*RevMan*): *version 7.2.0.* Available at http://revman.cochrane.org) and Stata (StataCorp. 2023. *Stata Statistical Software: Release 18*. College Station, TX: StataCorp LLC).

## Results

### Literature search

**Fig. 1:**
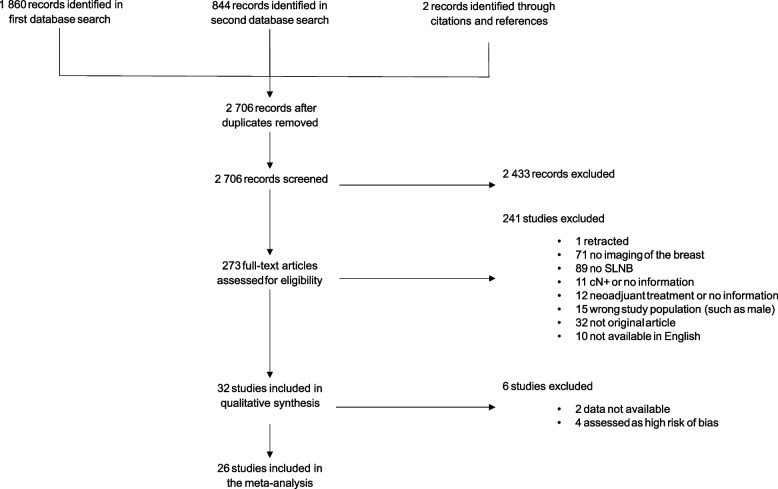
Flow chart of the systematic literature search and screening process

A total of 4 240 records were initially retrieved from the databases (Fig. 1). After the removal of duplicates, 1860 records remained. An updated search yielded 844 additional unique records. No additional records were identified through searches for gray literature; however, two additional records were identified by searching the reference list and citations of the included studies. The screening of titles and abstracts resulted in the exclusion of 2433 records. Full-text screening was performed on the remaining 273 records to assess their eligibility, resulting in the exclusion of 241 records. The records were excluded for the following reasons: retracted articles (*n* = 1) [27], articles without breast imaging (*n* = 71) [10, 12, 16, 28–95], studies in which SLNB was not performed for all patients (*n* = 89) [13–15, 96–181], studies including clinically node-positive patients or no information about clinical node status (*n* = 11) [182–192], studies including patients who received neoadjuvant treatment or no information about neoadjuvant treatment (*n* = 12) [193–204], studies with the wrong study population (*n* = 15) [205–219], unoriginal articles (*n* = 32) [220–251], and articles not available in English (*n* = 10) [252–261]. The remaining 32 studies [262–293] were included in the systematic review.

### Risk of bias assessment

**Fig. 2:**
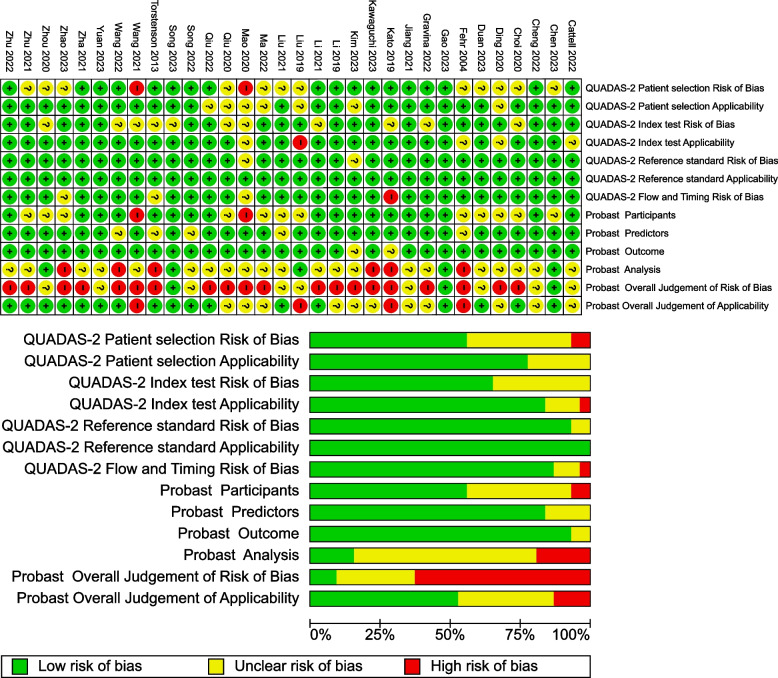
Summary of the bias assessments using QUADAS-2 and PROBAST and for individual studies. Quality Assessment of Diagnostic Accuracy Studies 2 (QUADAS-2), Prediction Model Risk of Bias Assessment Tool (PROBAST)

All 32 included articles were assessed for bias using the QUADAS-2 tool and PROBAST (Fig. 2). The results of the bias assessment with QUADAS-2 were classified into three categories: low risk of bias (*n* = 9) [264, 269, 271, 273, 275, 283, 288, 289, 292], unclear risk of bias (*n* = 19) [262, 263, 265–268, 270, 274, 276, 278, 279, 281, 282, 284, 285, 287, 290, 291, 293], and high risk of bias (*n* = 4) [272, 277, 280, 286]. The four studies assessed as having a high risk of bias were excluded from the meta-analysis. Studies were classified as having a low risk of bias (*n* = 3) [263, 269, 284], unclear risk of bias (*n* = 8) [262, 264, 267, 271, 278, 283, 288, 291], or high risk of bias (*n* = 21) [265, 266, 268, 270, 272–277, 279–282, 285–287, 289, 290, 292, 293] based on PROBAST assessments. The null hypothesis of equal bias assessments for the two tools could be rejected (*p* < 0.001), indicating that more studies were assessed as having a high risk of bias using the PROBAST tool.

### Data analysis

The study characteristics are presented in Table 1, and the patient characteristics are detailed in Supplementary 3, Table 1. Among the included studies, published from 2004 to 2023, 21 were published in China [263, 264, 267, 269, 271, 275, 276, 278–283, 286–293]. The remaining 11 studies were published in the USA (*n* = 4) [262, 266, 277, 285], South Korea (*n* = 3) [265, 274, 284], Japan (*n* = 2) [272, 273], Switzerland (*n* = 1) [268], and Italy (*n* = 1) [270]. The proportion of patients with pN + breast cancer ranged from 13 to 55%, and the studies included a total of 11,464 participants. The following five imaging modality classes were included: US (*n* = 8) [271, 278, 282, 285, 289–292], MRI (*n* = 17) [262, 263, 265, 266, 269, 270, 272, 273, 276, 277, 279–281, 283, 286, 287, 293], DM (*n* = 1) [288], PET‒CT (*n* = 1) [268], and multiple modalities (*n* = 5) [264, 267, 274, 275, 284]. The categories of multiple modalities included at least two of the following imaging modalities: US, MRI, DM, PET‒CT, and DBT. The technical aspects of each imaging modality varied between studies. Among the eight studies that used only US, four included both breast and axillary US [278, 282, 290, 291]. Seventeen studies relied exclusively on imaging features [263, 264, 268–274, 276, 279, 280, 282, 287, 288, 291, 293], whereas 15 combined imaging with clinical variables in the prediction model [262, 265–267, 275, 277, 278, 281, 283–286, 289, 290, 292]. CIs for AUCs were not reported in 14 studies [262–264, 266, 270, 272, 275–279, 287, 292, 293]. Additionally, one study did not report an AUC [268], and another did not provide sufficient data (CI or SE, and pN + proportion) for calculating CIs [276]. A comparison of the reported and calculated 95% CIs for the studies included in the meta-analysis that reported AUCs with CIs and/or SEs is presented in Supplementary 3, Fig. 1. Moreover, the imaging modality subgroup characteristics are presented in Table 2. The total number of patients included in the subgroups ranged from 3136–3926, with the most patients in the MRI subgroup.

**Table 1:** Baseline characteristics of the included studies

Study (author, year, country)	Study design	N in total	N in training	pN + (%) in training	Imaging modality included in the study	Method of feature extraction	Clinical predictors in model	Preoperative variables	Validation	Calibration	AUC
Cattell, 2022, USA	Retrospective	198	163	34%	DCE-MRI	VGG-16, manual	Yes	No	Internal	No	0.83^b^
Chen, 2023, China	Retrospective	988	648	27%	DCE-MRI	ResNet18, manual	No	Yes	Internal + external	No	0.89^c^
Cheng, 2022, China	Retrospective	208	138	47%	DM, DBT, DCE- and DWI-MRI	PyRadiomics toolkit, semi-manual with ITK-SNAP	No	Yes	Internal	No	0.83^b^
Choi, 2020, South Korea	Retrospective	309	309	13%	DCE-MRI	Kinetic curve, manual	Yes	Yes	No	No	0.84^a^
Ding, 2020, USA	Retrospective	162	162	34%	DCE-MRI	LIFEx + MatLab radiomics, manual with MRIcron	Yes	No	Internal	No	0.85^b^
Duan, 2023, China	Retrospective	326	162	41%	DM, BUS	PyRadiomics toolkit, semi-manual with 3DSlicer	Yes	Yes	Internal + external	Yes	0.84^c^
Fehr, 2004, Switzerland	Prospective	24	24	42%	FDG-PET-CT	Manual, manual	No	Yes	No	No	-
Gao, 2023, China	Retrospective	941	825	41%	DCE- and DWI-MRI	RCNet, semi-manual with 3DSlicer	No	Yes	Internal + external	No	0.85^c^
Gravina, 2022, Italy	Retrospective	155	155	17%	DCE-MRI	2DS-NET, automatic two-dimensional slice	No	Yes	Internal	No	0.78^b^
Jiang, 2021, China	Retrospective	239	190	33%	BUS	Manual + MatLab radiomics, manual	No	Yes	Internal	Yes	0.90^b^
Kato, 2019, Japan	Retrospective	44	44	27%	DWI-MRI	Manual, manual	No	Yes	No	No	0.78^a^
Kawaguchi, 2023, Japan	Retrospective	120	120	29%	DCE-MRI	Manual, no segmentation	No	Yes	No	No	0.92^a^
Kim, 2023, South Korea	Retrospective	964	603	21%	BUS, AUS, MRI	Manual, manual	No	Yes	Internal	Yes	0.65^b^
Li, 2019, China	Retrospective	397	397	50%	BUS, DCE-MRI	Manual, no segmentation	Yes	Yes	Internal	Yes	0.77^b^
Li, 2021, China	Retrospective	197	132	42%	DCE-MRI	Philips Radiomics Tool, manual with Philips Radiomics Tool	No	Yes	Internal	Yes	0.89^b^
Liu, 2019, USA	Retrospective	163	109	34%	DCE-MRI	LIFEx + MatLab radiomics, manual with MRIcron	Yes	No	Internal	No	0.87^b^
Liu, 2021, China	Retrospective	701	519	38%	BUS, AUS	Manual, manual	Yes	Yes	Internal + external	No	0.83^c^
Ma, 2022, China	Retrospective	142	100	37%	DCE-MRI	PyRadiomics toolkit, automatic 3D U-NET segmentation	No	Yes	Internal	No	0.82^b^
Mao, 2020, China	Retrospective	296	200	50%	DCE-MRI	Artificial Intelligence Kit software, manual	No	Yes	Internal	Yes	0.79^b^
Qiu, 2020, China	Retrospective	196	141	32%	BUS, AUS	Manual + PyRadiomics toolkit, semi-automated with 3DSlicer	No	Yes	Internal	No	0.76^b^
Qiu, 2022, China	Retrospective	100	71	55%	MRI	PyRadiomics toolkit, manual	Yes	Yes	Internal	Yes	0.79^b^
Song, 2022, China	Retrospective	432	296	36%	DCE-MRI	MR Radiomics platform, semi-automated with MR Radiomics platform	Yes	Yes	Internal	Yes	0.87^b^
Song, 2023, South Korea	Retrospective	948	402	20%	BUS, AUS, DM, MRI	Manual, manual	Yes	Yes	Internal + external	Yes	0.77^c^
Torstenson, 2013, USA	Retrospective	401	401	20%	BUS	Manual, no segmentation	Yes	No	No	Yes	0.77^a^
Wang, 2021, China	Case–control, retrospective	186	186	50%	DCE-MRI	Histogram analysis, manual	Yes	Yes	Internal	Yes	0.84^b^
Wang, 2022, China	Retrospective	202	140	54%	DWI-MRI	AMni-Kinetics software, semi-automated with 3DSlicer	No	Yes	Internal	No	0.82^b^
Yuan, 2023, China	Retrospective	649	487	41%	DM	Manual, no segmentation	No	Yes	Internal	Yes	0.83^b^
Zha, 2021, China	Retrospective	452	318	29%	BUS	PyRadiomics toolkit, manual	Yes	No	Internal	Yes	0.83^b^
Zhao, 2023, China	Retrospective	199	159	33%	BUS, AUS	PyRadiomics toolkit, semi-automated with 3DSlicer	Yes	Yes	Internal	No	0.79^b^
Zhou, 2020, China	Retrospective	834	756	50%	BUS, AUS	Inception V3, no segmentation	No	Yes	Internal + external	No	0.89^c^
Zhu, 2021, China	Retrospective	177	123	46%	DCE- and DWI-MRI	Darwin Scientific Research Platform, automated Darwin Scientific Research Platform	No	Yes	Internal	No	0.88^b^
Zhu, 2022, China	Prospective	114	114	48%	BUS, CE-elastography	Manual, manual	Yes	Yes	No	Yes	0.80^a^

*pN + *Pathologically node positive, *AUC *Area under the curve, *BUS *Breast ultrasound, *AUS *Axillary ultrasound, *CE *Contrast-enhanced, *DCE *Dynamic contrast-enhanced, *DWI *Diffusion-weighted, *MRI *Magnetic resonance imaging, *DM* Digital mammography, *DBT *Digital breast *tomosynthesis, FDG *Fluorodeoxyglucose, *PET* Positron emission tomography, *CT* Computed tomography, *VGG-16* Visual Geometry Group 16 (VGG-16), *ResNet18* Residual Network 18, *RCNet* Related context-driven network, *2DS-NET *Two-dimensional slice network, *3D U-NET *Three-dimensional U-shaped network

^a^Training

^b^Internal validation

^c^External validation

**Table 2:** Imaging modality subgroup characteristics

Imaging modality	Pooled AUC (95% CI)	N studies	Total sample size (N)	Validation type
Ultrasound	0.81 (95% CI 0.78–0.84)	8	3136	No = 1 Internal = 5 External = 2
Magnetic Resonance Imaging	0.85 (95% CI 0.82–0.87)	12	3926	No = 2 Internal = 8 External = 2
Other/mixed	0.78 (95% CI 0.72–0.83)	6	3492	Internal = 4 External = 2

*AUC *Area under the curve, *CI* Confidence interval

### Heterogeneity analyses

**Fig. 3:**
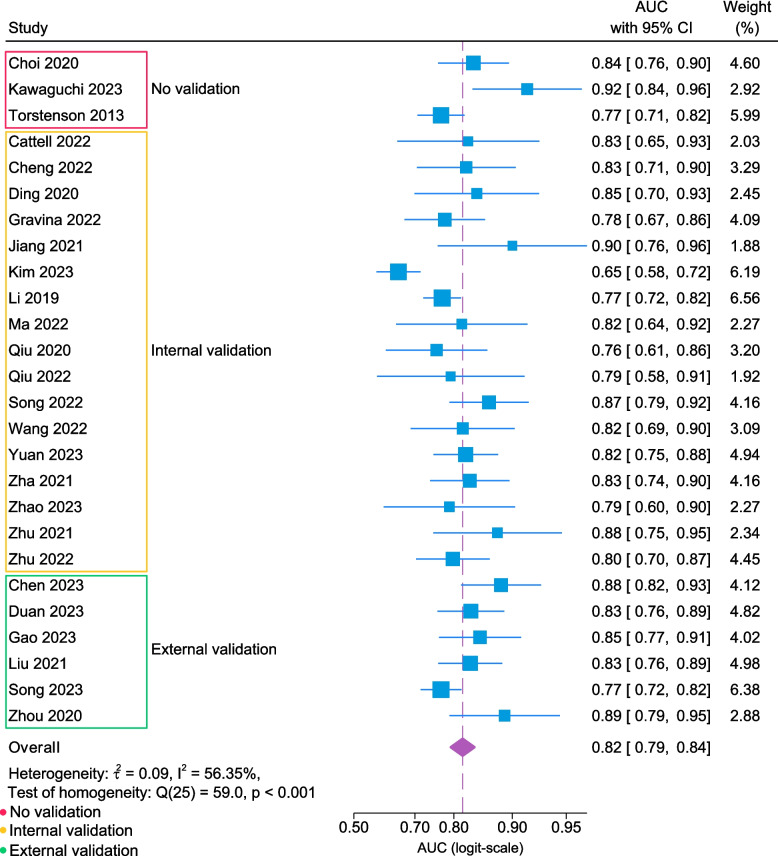
Forest plot summarizing model performance and heterogeneity (*n* = 26). Area under the curve (AUC), confidence interval (CI)

**Fig. 4:**
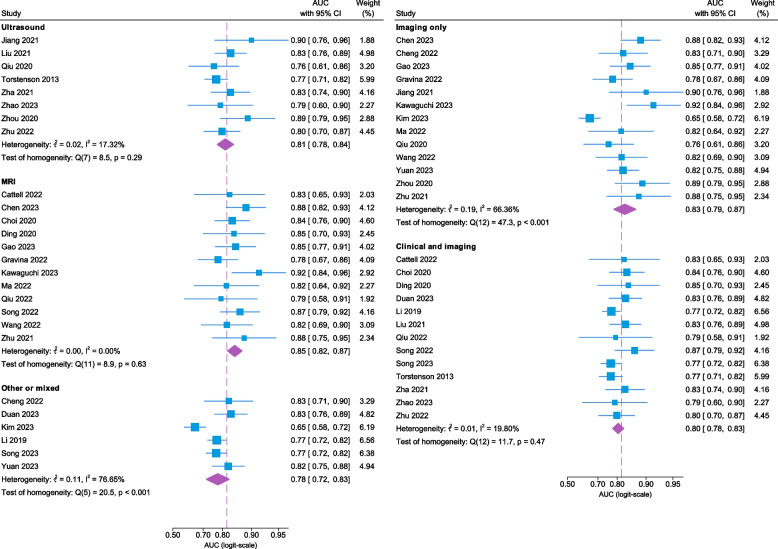
Forest plot stratified by subgroups summarizing model performance and heterogeneity (*n* = 26). Abbreviations: area under the curve (AUC), confidence interval (CI)

Owing to the missing information in the two studies and the exclusion of the four studies assessed as having a high risk of bias, only 26 [262–267, 269–271, 273–275, 278, 279, 281–285, 287–293] of the 32 studies were included in the meta-analysis. The performance of the prediction models was visualized using a forest plot (Fig. 3) also demonstrating the pooled AUC of 0.82 (95% CI 0.79–0.84). Heterogeneity analyses revealed an I^2^ of 56.4%, a Tau^2^ of 0.09, and a Cochran’s Q(25) of 59.0 (*p* < 0.001). Additionally, a subgroup analysis (Fig. 4) yielded pooled AUCs of 0.81 (95% CI 0.78–0.84), 0.85 (95% CI 0.82–0.87) and 0.78 (95% CI 0.72–0.83) for US, MRI, and other/mixed modalities, respectively. The corresponding heterogeneity metrics for each subgroup were as follows: US: I^2^ 17.3%, Tau^2^ 0.02, and Cochran’s Q(7) 8.5 (*p* = 0.29); MRI: I^2^ 0.00%, Tau^2^ 0.00, and Cochran’s *Q*(11) 8.9 (*p* = 0.63); and other/mixed modalities: I^2^ 76.7%, Tau^2^ 0.11, and Cochran’s *Q*(5) 20.5 (*p* < 0.001). Further stratification by the included data types (Fig. 4) yielded pooled AUCs of 0.83 (95% 0.79–0.87) and 0.80 (95% CI 0.78–0.83) for the imaging-only and clinical + imaging models. The corresponding heterogeneity metrics were as follows: imaging-only models: I^2^ 66.4%, Tau^2^ 0.19, and Cochran’s *Q* 47.3 (*p* < 0.001); and clinical + imaging models: I^2^ 19.8%, Tau^2^ 0.01, and Cochran’s Q 11.7 (*p* = 0.47).

**Fig. 5:**
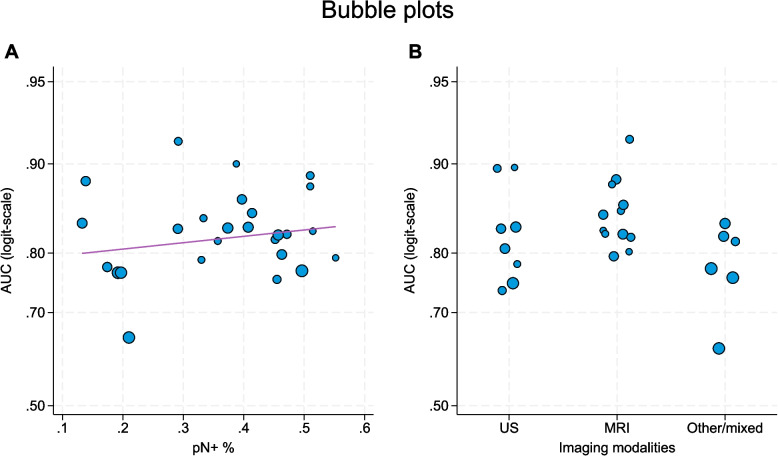
Bubble plots of **A** pN+% and **B** imaging modality. The fitted line in (**A**) represents the expected increase in logit AUC as function of pN+% estimated using meta-regression. The sizes of the bubbles are inversely proportional to the variances of the AUC estimates. Area under the curve (AUC), magnetic resonance imaging (MRI), pathologically node positive (pN+), ultrasound (US)

Univariable meta-regression analysis (Table 3) revealed evidence for an association between study-specific AUCs and imaging modality (*p* = 0.015), where MRI-based studies displayed higher AUCs. Additionally, an association with the AUC was displayed by single versus multiple imaging modalities (*p* = 0.005), where a single modality was associated with a higher AUC, and model calibration assessment upon validation (yes/no) (*p* = 0.026), where studies in which model calibration assessment was performed displayed lower AUCs. No evidence for an association with the AUC was found for the country of publication, year of publication, total study sample size, proportion of pN + patients, imaging of the axilla, ROI preprocessing method, method of feature extraction, feature selection, type of data, or type of validation (Table 3). A bubble plot illustrating the associations between the AUC and the proportion of pN + patients (Fig. 5A) and the choice of imaging modality (Fig. 5B) revealed a tendency toward increasing AUCs with increasing proportions of pN + patients and revealed that MRI-based models had higher AUCs than studies using US and other/mixed modalities. Additionally, meta-regression analyses of the QUADAS-2 tool (*p* = 0.467) and PROBAST assessments (*p* = 0.137), including all studies with AUCs and 95% CIs (*n* = 30), demonstrated no evidence of contributing to heterogeneity. Moreover, the multivariable meta-regression analysis including imaging modality, type of validation, total study sample size, and proportion of pN + patients, indicated that, compared with other imaging modalities, MRI is independently associated with a higher AUC (*p* < 0.001).

**Table 3:** Meta-regression analysis of the associations between the AUC and covariables

Variable	AUC	95% CI	*P*
**Country of publication**			0.070
China	0.832	0.804–0.857	
Other	0.787	0.742–0.827	
Publication year^*^			0.600
2013	0.796	0.688–0.874	
2018	0.811	0.766–0.848	
2023	0.824	0.792–0.852	
**Imaging**			0.015
Ultrasound	0.816	0.774–0.852	
MRI	0.849	0.816–0.877	
Other/mixed	0.774	0.730–0.813	
**Imaging focus**			0.989
Breast only	0.819	0.767–0.863	
Breast + axilla	0.819	0.789–0.846	
**Single versus multiple imaging modalities**			0.005
Single	0.834	0.810–0.856	
Multiple	0.764	0.715–0.808	
**Combined model**			0.535
Imaging only	0.828	0.790–0.860	
Clinical + imaging	0.812	0.775–0.844	
**Preprocessing method**			0.948
NA	0.826	0.769–0.871	
Manual	0.811	0.768–0.847	
Semi-automated	0.828	0.775–0.871	
Automated	0.821	0.722–0.890	
**Feature extraction method**			0.103
Manual	0.785	0.746–0.819	
Semi-automated	0.836	0.799–0.868	
Automated	0.851	0.799–0.891	
Mixed	0.821	0.699–0.900	
**Feature selection method**			0.233
Deep learning	0.851	0.794–0.894	
Other	0.814	0.785–0.841	
**Validation**			0.636
No validation	0.835	0.764–0.888	
Internal	0.805	0.769–0.836	
External	0.840	0.793–0.878	
**Calibration assessment**			0.026
No	0.842	0.811–0.869	
Yes	0.792	0.757–0.823	
Sample size in total^*^ (n)			0.436
100	0.830	0.791–0.862	
500	0.818	0.792–0.841	
1000	0.802	0.745–0.848	
Proportion of pN + ^*^ (%)			0.367
20	0.806	0.765–0.841	
25	0.819	0.794–0.842	
50	0.832	0.793–0.864	
**QUADAS-2**^**a**^			0.467
Low risk of bias	0.837	0.798–0.869	
Unclear risk of bias	0.807	0.774–0.836	
High risk of bias	0.823	0.752–0.878	
**PROBAST**^**a**^			0.168
Low risk of bias	0.829	0.766–0.878	
Unclear risk of bias	0.847	0.807–0.881	
High risk of bias	0.802	0.771–0.829	

*AUC *Area under the curve, *CI *Confidence interval, *MRI *Magnetic resonance imaging, *pN + *Pathologically node positive, *QUADAS-2 *Quality Assessment of Diagnostic Accuracy Studies 2, *PROBAST *Prediction Model Risk of Bias Assessment Tool

^*^Continuous variable: AUC values and 95% CIs shown for selected values

^a^Including all 30 studies

### Sensitivity analysis

Sensitivity analysis was performed by excluding studies with a high risk of bias according to the QUADS-2 assessment. No significant changes in performance or heterogeneity were observed (Supplementary 3 Fig. 2).

### Publication bias assessment

**Fig. 6:**
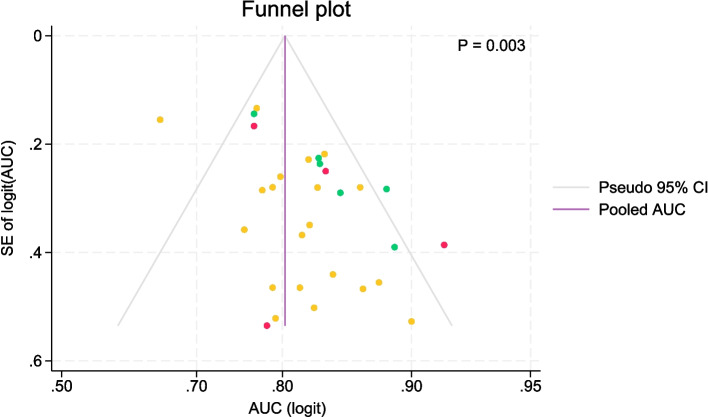
Funnel plot of publication bias (*n* = 30) stratified by degree of validation. High AUC estimates with high SEs (lower right corner) were more common than low AUC estimates with broad SEs (lower left corner). Area under the curve (AUC), confidence interval (CI), standard error (SE)

The funnel plot (Fig. 6) shows study specific AUC estimates plotted versus the corresponding SEs on a logit scale. The pooled AUC estimate and approximate 95% CIs around this estimate for different SEs are shown for comparison. High AUC estimates for small studies, which have larger SEs (lower right corner), were more common than low AUC estimates (lower left corner) (*p* = 0.003).

## Discussion

In this systematic review and meta-analysis, we addressed the potential of different breast imaging modalities for the prediction of SLN status in patients with early breast cancer who are eligible for SLNB. The systematic literature search and screening resulted in the inclusion of 32 studies [262–293], 26 of which were included in the meta-analysis [262–267, 269–271, 273–275, 278, 279, 281–285, 287–293]. The heterogeneity in the AUC was substantial across all the studies and in subgroup analyses, except for the analysis of MRI-based studies, which exhibited homogeneity. Compared with other studies, MRI-based studies, single-modality studies, and studies in which model calibration assessment upon validation was not performed presented higher AUCs in the meta-regression analyses. The sensitivity analysis revealed no significant changes in overall performance or heterogeneity when studies assessed as having a high risk of bias according to the QUADAS-2 were excluded. Additionally, the funnel plot suggested potential publication bias, with underrepresentation of studies with high SEs and low AUCs.

The bias assessment was performed using both the QUADAS-2 tool and PROBAST, each addressing different sources of bias and applicability concerns. The QUADAS-2 tool was developed primarily for diagnostic tests [25], whereas PROBAST was developed for prediction models and focuses on the methodology of predictor interpretation and selection, model development, and validation [26]. Sensitivity analysis was performed by excluding studies assessed as having a high risk of bias according to QUADAS-2; the exclusions did not affect the overall results. Moreover, the test of symmetry indicated that significantly more studies were assessed as having a high risk of bias via PROBAST. However, the meta-regression analysis of the bias assessments, including all studies with available data [262–267, 269–275, 277–293] (*n* = 30), revealed no association with the AUC, indicating that bias assessed by these tools cannot explain the observed heterogeneity between studies.

A comparison between the reported and calculated CIs for studies that provided AUC values with CIs revealed strong agreement. This suggests that the approximation formula is likely valid and can be reliably applied to studies that did not report a CI or SE for the AUC.

The analysis of the overall AUC heterogeneity confirmed substantial variability between studies, as expected when studies with different inclusion criteria, prevalence rates of the outcome, image modalities, clinical predictors, and modeling strategies were compared. Given this high degree of heterogeneity and the fact that the majority of studies did not perform an external validation, pooled estimates of AUCs in the forest plots should be interpreted with caution. Additionally, the varying degrees of validation, with external validation often performed in smaller cohorts, could influence the SEs and CIs, and the interpretation of the results. A lower AUC, compared with that obtained in the training cohort, is generally expected upon external validation. However, the meta-regression analysis of the prediction model validation showed weak evidence of an association with the AUC.

The forest plot divided into imaging modality subgroups revealed heterogeneity within the US subgroups and other/mixed subgroups, whereas the MRI subgroup was more homogeneous. MRI was shown to be associated with greater AUC in the univariable meta-regression, as shown in the bubble plot, as well as when adjusted for the type of validation, proportion of pN + , and total study sample size, indicating that MRI might be a superior imaging method for the prediction of SLN status in patients with early breast cancer. Although the MRI subgroup yielded an acceptable pooled AUC of 0.85 (95% CI 0.82–0.87), this finding must be interpreted in the clinical context and account for the consequences of false-positive and false-negative predictions. Additionally, the homogeneity of the MRI group indicates that differences in technical aspects, such as the strength of the magnetic field (Tesla) and the use of contrast, had limited effects on the performance of the models. However, the heterogeneity within the two other subgroups renders the comparison with MRI unreliable. The US subgroup consists of studies that use breast US alone or in combination with AUS, whereas the other/mixed group comprises several imaging modalities. The univariable meta-regression did not, however, identify an association between performance and the use of breast images alone or in combination with axillary images. Moreover, variations in clinical imaging practices may have led to patient selection on the basis of unspecified criteria, introducing potential selection bias that could influence the results of the meta-analysis. An example is the variability in recommendation of AUS as part of the clinical work-up [1, 2, 4], although AUS offers several advantages such as being a direct assessment of the lymph nodes rather than relying on indirect breast features. Furthermore, the meta-regression analysis of a single imaging modality versus multiple imaging modalities indicated that the use of a single imaging modality was associated with a greater AUC. Given the evident heterogeneity in the included imaging modalities between studies in the multiple imaging subgroup, the results of the meta-regression were expected and should be interpreted with caution.

The inclusion of both imaging features and clinical variables such as histological grade, the proliferation marker Kiel 67 (Ki67), lymphovascular invasion, age, and various lymphocyte biomarkers, in the prediction models was not identified as a source of heterogeneity in the meta-regression analysis. This finding indicates that the addition of clinical variables to imaging features did not improve the overall AUC. Given that many of these predictors are well-established as key factors for predicting SLN status [10, 11], this finding is unexpected and warrants further investigation. This could be due to overfitting, although the meta-regression did not indicate that the type of validation was associated with the AUC and that several studies included a well calibrated external validation, suggesting that the models in general were not overfit. Another potential explanation could be the method used for integrating clinical variables, such as initially developing a model using imaging and then evaluating the addition of clinical variables rather than incorporating the variables in the first selection process.

The proportion of pN + patients varies substantially between different populations, as illustrated by the diversity in the included studies [262, 265, 281, 284, 288]. From a statistical perspective, these differences could affect model performance and reduce the generalizability of models to a population with a different proportion of pN + patients. Although the meta-regression results suggest that the proportion of pN + patients was not a major contributor to heterogeneity in the included studies, the bubble plot illustrates a tendency toward an increasing AUC with an increasing proportion of pN + patients, which was expected. To increase the generalizability of prediction models in different populations, the proportion of pN + patients could be included in the prediction model, as demonstrated by Meretoja et al. [294].

The methods used for preprocessing, feature extraction, feature selection, and model development and validation in the included studies presented high variability. For the purpose of the systematic review, studies were grouped into manual, semiautomated (i.e., predefined radiomic features), and automated (i.e., allowing AI to autonomously construct, often imperceptible, patterns and features). There are, however, hybrid approaches such as extracting and selecting standardized radiomic features while training an AI model to use the selected features to predict nodal status. A binary classification of AI/non-AI could be considered, but because of the diversity of how and at which stage AI was defined and employed in various studies it was decided that this would be too arbitrary and would contribute little to the analysis. Previous studies have indicated that automated methods such as deep learning could outperform radiomics [18, 295]. However, the meta-regression analysis identified no apparent association between the method used for any of the categories listed above, except for model calibration assessment upon validation and the AUC. Given the diverse range of approaches, methods have been categorized to enable analyses, resulting in broad groups. Thus, a potential difference between specific methods could be difficult to detect. This finding further highlights the heterogeneity between studies, the diversity of methods that can be deployed to solve the same task with similar results, and the necessity of additional studies to enable more homogenous comparisons. Importantly, the MRI subgroup showed homogeneity in performance regardless of differences in methods for preprocessing, feature extraction, feature selection and model development, indicating that high AUCs can be achieved in this subgroup regardless of the choice of methods.

The funnel plot illustrates the lack of studies with larger SEs and lower AUCs, suggesting potential publication bias, which was confirmed by Egger’s test (*p* = 0.003). This was expected and could for example be explained either by authors not submitting, so-called, negative results in smaller studies or by journals rejecting such manuscripts. Such bias could lead to biased results and incorrectly high pooled AUCs.

A previous meta-analysis by Deng et al. [20] suggested that contrast-enhanced (CE) US could serve as a noninvasive alternative to SLNB, as it yielded a pooled AUC of 0.94. However, the study included both patients with confirmed breast cancer who underwent either upfront SLNB or SLNB after neoadjuvant therapy, making the interpretation of the results complex from a clinical perspective. Nonetheless, CE-US and US should be further evaluated for more defined patient populations. Another meta-analysis by Zhang et al. [18] reported that the pooled AUC of dynamic contrast enhanced (DCE) MRI was 0.89 (95% CI 0.86–0.91). Subgroup analyses revealed that manual ROI preprocessing and a stronger magnetic field, among other factors, could improve diagnostic performance, whereas our results indicate that the MRI subgroup was more homogeneous regardless of varying technical differences between studies. A systematic review by Gong et al. [17] reported that radiomics models based on various imaging modalities represent promising noninvasive alternatives to surgical axillary staging, which is in line with our results. Their meta-regression and subgroup analyses revealed that the imaging modality, method of modeling, country of publication and combining imaging data with clinical data contributed to heterogeneity. In contrast, our analysis identified only the imaging modality as a significant source of heterogeneity. However, similar to the studies by Deng et al. [20] and Zhang et al. [18], Gong et al. included a heterogeneous cohort of patients with breast cancer of any stage who underwent SLNB or ALND, which complicates the clinical interpretation as neoadjuvant treatment substantially influences SLN status [296]. In the context of current guidelines for breast cancer treatment and management of the axilla [1, 2], differentiating between patient subgroups is essential for supporting more individualized decision-making. This highlights the need for research on clinically relevant subgroups.

There are many strengths of this systematic review. To the authors’ knowledge, this is the first systematic review and meta-analysis evaluating prediction models for SLN status using breast imaging specifically in patients with early breast cancer who are eligible for SLNB. First, the study investigated the performance of noninvasive prediction models in a well-defined patient population eligible for SLNB, providing insights into further research on the specific population. Second, several imaging modalities, preprocessing techniques, feature extraction and selection methods, and model development approaches are comprehensively compared. However, this study also has limitations. Considerable heterogeneity was observed across studies, except in the MRI subgroup, thus limiting the reliability of the overall pooled AUC estimate as well as estimates for the other subgroups. Additionally, the included AUCs were derived from datasets with varying degrees of validation, potentially overestimating the pooled results. Furthermore, only one study included a prediction model using DM [288] and no studies included CT or DBT only, thus making the study somewhat incomplete from a radiological perspective. Moreover, only two prospective studies were included [268, 292], one of which did not provide an AUC [268] and could thus not be included in the meta-analysis. Therefore, more prospective studies are needed to validate the findings of this study. Furthermore, the proportion of pN + patients in the included studies varied considerably from 13–55%, which could reflect differences in screening programs. Given that the proportion of pN + patients can directly influence model performance, this may affect the generalizability of the results, underscoring the necessity of additional studies in other populations. Other potential differences in the study population, not captured in the available data, could also have contributed to the observed heterogeneity. Additionally, while the screening, data collection, and bias assessments were conducted by two independent reviewers, only one of the reviewers (CR) participated in all of the steps for all records. Finally, the updated literature search was performed in March 2024 and more novel publications were therefore not considered for inclusion. In conclusion, this is the first study to evaluate prediction models with several imaging modalities and methods of model development, specifically in women with early breast cancer. However, the substantial heterogeneity between studies regarding the proportion of pN + , methods for data extraction, model development, and validation may affect pooled AUC estimates and the generalizability of the results.

## Conclusions

This systematic review and meta-analysis revealed that prediction models using breast imaging could be potential noninvasive alternatives to SLNB in patients with early breast cancer. The MRI subgroup showed homogeneity and a high pooled AUC, indicating that prediction models using MRI could be superior to other imaging modalities for these patients. To facilitate the clinical implementation of prediction models, prospective and more robust studies are needed to evaluate the potential of different imaging modalities and modeling strategies.

## Supplementary Information

Supplementary Material 1.

Supplementary Material 2.

Supplementary Material 3.

## Data Availability

All the data included has been previously published. The template for data collection, data extracted from included studies, datasets established for the analysis, and code for statistical analyses is available from the corresponding author on reasonable request.

## Abbreviations

AI: Artificial intelligence
ALND: Axillary lymph node dissection
AUS: Axillary ultrasound
AUC: Area under the curve
BUS: Breast ultrasound
CHARMS: CHecklist for critical Appraisal and data extraction for systematic Review of prediction Modeling Studies
cN0: Clinically node-negative
CT: Computed tomography
CI: Confidence interval
CE: Contrast-enhanced
CINAHL: Cumulative Index to Nursing and Allied Health Literature
DWI: Diffusion-weighted
DIVA: Digitala vetenskapliga arkivet
DBT: Digital breast tomosynthesis
DM: Digital mammography
DCE: Dynamic contrast-enhanced
FDG: Fluorodeoxyglucose
INSEMA: Intergroup Sentinel Mamma study
Ki67: Kiel 67
LASSO: Least absolute shrinkage and selection operator
MRI: Magnetic resonance imaging
PROSPERO: International Prospective Register of Systematic Reviews
PET: Positron emission tomography
pN +: Pathologically node positive
PROBAST: Prediction Model Risk of Bias Assessment Tool
PRISMA: Preferred Reporting Items for Systematic Reviews and Meta-analysis
ROI: Region of interest
SLN: Sentinel lymph node
SLNB: Sentinel lymph node biopsy
SOUND: Sentinel Node vs Observation After Axillary Ultra-Sound
SE: Standard error
US: Ultrasound
QUADAS-2: Quality Assessment of Diagnostic Accuracy Studies 2
