# Supplementary material for: Prediction of sentinel lymph node status in patients with early breast cancer using breast imaging as an alternative to surgical staging—a systematic review and meta-analysis
Source: Syst Rev. 2025 Nov 25;14:246. doi: 10.1186/s13643-025-03005-9 (PMC12687516; doi:10.1186/s13643-025-03005-9)
Supplement: Supplementary file 2 — Supplementary Material 2. [file 13643_2025_3005_MOESM2_ESM.pdf]

Krister Aronsson, Medicinska fakulteten

LUND UNIVERSITY
Faculty of Medicine

SYSTEMATIC LITERATURE SEARCH STRATEGY

SEARCH TERMS

BREAST NEOPLASMS/CANCER/TUMORS, MAMMARY NEOPLASMS/CANCER/TUMORS, PREDICTION, LYMPHATIC METASTASIS, METASTASES, NODE POSITIVITY, SENTINEL LYMPH NODE, AXILLARY LYMPH NODE, MAMMOGRAPHY, ULTRASONOGRAPHY, ULTRASOUND, COMPUTED TOMOGRAPHY, MAGNETIC RESONANCE IMAGING, ARTIFICIAL NEURAL NETWORK, CONVENTIONAL NEURAL NETWORK, MACHINE LEARNING, DEEP LEARNING, TRANSFER LEARNING, NOMOGRAMS, LOGISTIC REGRESSION, MATHEMATICAL MODELS, RADIOMICS, CLINICOPATHOLOGICAL, BREAST IMAGING REPORTING AND DATA SYSTEMS, CLINICALLY NODE NEGATIVE, CLINICALLY NEGATIVE LYMPH

SCHEDUALS

20211228
PubMed
#1
breast neoplasms[Mesh]
=317663

#2
breast neoplasm*[Title/Abstract] OR breast cancer[Title/Abstract] OR breast tumo*[Title/Abstract] OR mammary cancer[Title/Abstract] OR mammary neoplasm*[Title/Abstract] OR mammary

tumo*[Title/Abstract] OR BC[Title/Abstract] OR breastcancer[Title/Abstract] OR breasttumo*[Title/Abstract] OR breastneoplasm*[Title/Abstract]
=356329

#3
#1 OR #2
=445073

#4
predict*[Title/Abstract]
=1800257

#5
"Lymphatic Metastasis"[Mesh]
=94083

#6
node positivity[Title/Abstract] OR metastasis[Title/Abstract] OR metastases[Title/Abstract] OR SLN[Title/Abstract] OR sentinel lymph node*[Title/Abstract] OR staging[Title/Abstract] OR stage*[Title/Abstract] OR ALN[Title/Abstract] OR axillary lymph node*[Title/Abstract]
=1561204

#7
#5 OR #6
=1589253

#8
((("Mammography"[Mesh]) OR "Ultrasonography, Mammary"[Mesh]) OR "Magnetic Resonance Imaging"[Mesh]) OR "Tomography, X-Ray Computed"[Mesh]
=915382

#9
mammograph*[Title/Abstract] OR mammogram*[Title/Abstract] OR CT[Title/Abstract] OR computed tomograph*[Title/Abstract] OR US[Title/Abstract] OR ultrasound[Title/Abstract] OR ultrasonograph*[Title/Abstract] OR MRI[Title/Abstract] OR magnetic resonance imaging[Title/Abstract]
=1671327

#10
#8 OR #9
=2036011

#11
ML[Title/Abstract] OR "machine learning"[Title/Abstract] OR ANN[Title/Abstract] OR "artificial neural network*"OR "conventional neural network*"[Title/Abstract] OR CNN[Title/Abstract] OR "transfer learning"[Title/Abstract] OR "deep learning"[Title/Abstract] OR nomogram*[Title/Abstract] OR logistic

regression*[Title/Abstract] OR mathematical model*[Title/Abstract] OR radiomics[Title/Abstract]
=846531

#12
((("Machine Learning"[Mesh]) OR "Deep Learning"[Mesh]) OR "Nomograms"[Mesh]) OR "Logistic regression"[Mesh]
=42774

#13
#11 OR #12
=856543

#14
clinicopathological OR BI-RADs OR BI-RAD OR "breast imaging reporting and data system*"[Title/Abstract] OR “breast imaging-reporting and data system*"[Title/Abstract]
=107197

#15
#13 OR #14
=947875

#16
#10 AND #15
=101257

#17
clinically node-negative[Title/Abstract] OR clinically node negative[Title/Abstract] OR clinically negative lymph*[Title/Abstract]
=1151

#18
#16 OR #17
=102380

#19
#3 AND #4 AND #7 AND #18
=833 referenser

#20
#19 Filters: Female
=633

20211229
Embase

#1
'breast tumor'/exp OR 'breast cancer'/exp OR 'breast tumo*':ab,ti OR 'breast cancer':ab,ti OR 'breast neoplasm*':ab,ti OR breastcancer:ab,ti OR breasttumo*:ab,ti OR breastneoplasm*:ab,ti OR 'mammary cancer':ab,ti OR 'mammary tumo*':ab,ti OR 'mammary neoplasm*':ab,ti
=661678

#2
predict*:ab,ti
=2407430

#3
'lymph node metastasis'/exp OR 'node positivity' OR 'sentinel lymph node'/exp OR 'sentinel lymph node biopsy'/exp OR 'node positivity':ab,ti OR metastasis:ab,ti OR metastases:ab,ti OR 'sentinel lymph node*':ab,ti OR staging:ab,ti OR stage*:ab,ti OR sln:ab,ti OR aln:ab,ti OR 'axillary lymph node*':ab,ti
=2193244

#4
'mammography'/exp OR 'echomammography'/exp OR 'nuclear magnetic resonance imaging'/exp OR 'x-ray computed tomography'/exp OR mammograph*:ab,ti OR mammogram*:ab,ti OR mri:ab,ti OR 'magnetic resonance imaging':ab,ti OR ct:ab,ti OR 'computed tomograph*':ab,ti OR us:ab,ti OR ultrasonograph*:ab,ti OR ultrasound:ab,ti OR echograph*:ab,ti
=2909515

#5
'machine learning'/exp OR 'deep learning'/exp OR 'nomogram'/exp OR 'logistic regression analysis'/exp OR 'artificial neural network'/exp OR ml:ab,ti OR 'machine learning':ab,ti OR 'deep learning':ab,ti OR nomogram*:ab,ti OR ann:ab,ti OR 'artificial neural network*':ab,ti OR 'conventional neural network*':ab,ti OR cnn:ab,ti OR 'transfer learning':ab,ti OR transferlearning:ab,ti OR 'logistic regression*':ab,ti OR machinelearning:ab,ti OR radiomics:ab,ti OR 'mathematical model*':ab,ti
=2143463

#6
clinicopathological OR (((clincopatholigcal:ab,ti OR 'bi rad':ab,ti OR birads:ab,ti OR 'breast imaging reporting':ab,ti) AND 'data system*':ab,ti OR birad:ab,ti OR birads:ab,ti OR 'breast imaging-reporting':ab,ti) AND 'data system*':ab,ti)
=93467

#7
#5 OR #6
=2229995

#8
#4 AND #7
=244575

#9
'clinically node negative' OR 'clinically node-negative' OR 'clinically negative lymph' OR 'clinically node negative':ab,ti OR 'clinically node-negative':ab,ti OR 'clinically negative lymph*':ab,ti
=1954

#10
#8 OR #9
=246463

#11
#1 AND #2 AND #3 AND #10
=1509

#12
#11 AND [female]/lim
=1102

20220104
Web of science
#1
((TS=(breast neoplasms OR breast cancer OR breast tumors)) OR TI=(breast neoplasm* OR breast cancer OR breast tumo* OR mammary cancer OR mammary neoplasm* OR mammary tumo* OR breastcancer OR breastneoplasm* OR breasttumo*)) OR AB=(breast neoplasm* OR breast cancer OR breast tumo* OR mammary cancer OR mammary neoplasm* OR mammary tumo* OR breastcancer OR breastneoplasm* OR breasttumo*)
=934220

#2
(TI=(predict*)) OR AB=(predict*)
=4349474

#3
((TS=(lymphatic metastasis OR node positivity OR sentinel lymph node OR axillary lymph node)) OR TI=(node positivity OR metastasis OR metastases OR SLN OR sentinel lymph node* OR staging OR stage* OR ALN OR axillary lymph node*)) OR AB=(node positivity OR metastasis OR metastases OR SLN OR sentinel lymph node* OR staging OR stage* OR ALN OR axillary lymph node*)
=3458871

#4
((TS=(mammography OR ultrasonography OR "magnetic resonance imaging" OR computed tomography OR X-ray computed tomography)) OR TI=(mammograph* OR mammogram* OR computed tomograp* OR ultrasound OR CT OR US OR ultrasonograph* OR MRI OR magnetic resonance imaging)) OR AB=(mammograph* OR mammogram* OR computed tomograp* OR ultrasound OR CT OR US OR ultrasonograph* OR MRI OR magnetic resonance imaging)

=3875428

#5
((TS=(machine learning OR deep learning OR nomograms OR logistic regression)) OR TI=(ML OR "machine learning" OR "deep learning" OR ANN OR "artificial neural network*" OR CNN OR "conventional neural network*" OR nomogram* OR logistic regression OR mathematical model* OR radiomics)) OR AB=(ML OR "machine learning" OR "deep learning" OR ANN OR "artificial neural network*" OR CNN OR "conventional neural network*" OR nomogram* OR logistic regression OR mathematical model* OR radiomics)
=3184362

#6
((TS=(BI-RADs OR breast imaging reporting and data systems)) OR TI=(clinicopathological OR BI-RAD OR BI-RADs OR "breast imaging reporting and data system*")) OR AB=(clinicopathological OR BI-RAD OR BI-RADs OR "breast imaging reporting and data system*")
=81334

#7
#5 OR #6
=3260290

#8
#4 AND #7
=223840

#9
(TI=(clinically node negative OR clinically node-negative OR clinically negative lymph*)) OR AB=(clinically node negative OR clinically node-negative OR clinically negative lymph*)
=7229

#10
#8 OR #9
=238841

#11
#1 AND #2 AND #3 AND #10
=1477

#12
and Female (MeSH Headings)
=1003

Cochrane
#1

(breast neoplasm* OR breast cancer OR breast tumo* OR mammary neoplasm* OR mammary cancer OR mammary tumo* OR breastneoplasm* OR breastcancer OR breasttumo*):ti,ab,kw
=41115

#2
(predict*):ti,ab,kw
=105778

#3
(node positivity OR node positive OR node-positive OR sentinel lymph node* OR metastasis OR metastases OR staging OR stage* OR SLN OR ALN OR axillary lymph node*):ti,ab,kw
=199906

#4
(mammograph* OR echomammograph* OR mammogram* OR magnetic resonace imaging OR X-ray computed tomography OR computed tomography OR MRI OR US or ultrasound OR ultrasonograph*):ti,ab,kw
=121870

#5
(ML OR "machine learning" OR "deep learning" OR "transfer learning" OR nomogram* OR ANN OR artificial neural network* OR CNN OR conventional neural network* OR "logistic regression*" OR mathematical model* OR radiomics):ti,ab,kw
=158492

#6
(clinicopathological OR BI-RAD OR BI-RADs OR "breast imaging reporting and data system*" OR "breast imaging-reporting and data system*"):ti,ab,kw
=1469

#7
#5 OR #6
=159717

#8
#4 AND #7
=16165

#9
(clinically node negative OR clinically node-negative OR clinically negative lymph*):ti,ab,kw
=5135

#10
#8 OR #9
=21165

#11
#1 AND #2 AND #3 AND #10
=440
(1 review, 439 trials)
Begränsning till kön ej möjlig.

20220105
CINAHL
#1
(MH "Breast Neoplasms+") OR TI (breast neoplasm* OR breast cancer OR breast tumo* OR mammary cancer OR mammary neoplasm* OR mammary tumo* OR breastcancer OR breastneoplasm* OR breasttumo*) OR AB (breast neoplasm* OR breast cancer OR breast tumo* OR mammary cancer OR mammary neoplasm* OR mammary tumo* OR breastcancer OR breastneoplasm* OR breasttumo*)
=115846

#2
TI predict* OR AB predict*
=412506

#3
MM sentinel lymph node OR TI (node positivity OR node positive OR node-positive OR sentinel lymph node* OR SLN OR axillary lymph node* OR ALN OR staging OR stage*) OR AB (node positivity OR node positive OR node-positive OR sentinel lymph node* OR SLN OR axillary lymph node* OR ALN OR staging OR stage*)
=202604

#4
((MM "Mammography") OR (MH "Ultrasonography+") OR (MH "Magnetic Resonance Imaging+") OR (MH "Radiographic Image Enhancement+")) OR TI (mammograph* OR echomammograph* OR mammogram* OR magnetic resonace imaging OR X-ray computed tomography OR computed tomography OR MRI OR US or ultrasound OR ultrasonograph*):) OR AB (mammograph* OR echomammograph* OR mammogram* OR magnetic resonace imaging OR X-ray computed tomography OR computed tomography OR MRI OR US or ultrasound OR ultrasonograph*):)
=392744

#5
((MH "Machine Learning+") OR (MM "Deep Learning") OR (MH "Logistic Regression+")) OR TI (ML OR "machine learning" OR "deep learning" OR transfer learning OR ANN OR "artificial neural network*" OR CNN OR "conventional neural network*" OR nomogram* OR logistic regression OR mathematical model* OR radiomics) OR AB (ML OR "machine learning" OR "deep learning" OR transfer learning OR ANN OR "artificial neural network*" OR CNN OR "conventional neural network*" OR nomogram* OR logistic regression OR mathematical model* OR radiomics)
=322378

#6

TI (clinicopathological OR BI-RAD OR BI-RADs OR "breast imaging reporting and data system*" OR "breast imaging-reporting and data system*") OR AB (clinicopathological OR BI-RAD OR BI-RADs OR "breast imaging reporting and data system*" OR "breast imaging-reporting and data system*")
=8400

#7
#5 OR #6
=329986

#8
#4 AND #7
=26189

#9
TI (clinically node negative OR clinically node-negative OR clinically negative lymph*) OR AB (clinically node negative OR clinically node-negative OR clinically negative lymph*)
=934

#10
#8 OR #9
=27098

#11
#1 AND #2 AND #3 AND #10
=296

#12
#11
Narrow by SubjectGender: - female
=**229**
