# Supplementary material for: Prediction of sentinel lymph node status in patients with early breast cancer using breast imaging as an alternative to surgical staging—a systematic review and meta-analysis
Source: Syst Rev. 2025 Nov 25;14:246. doi: 10.1186/s13643-025-03005-9 (PMC12687516; doi:10.1186/s13643-025-03005-9)
Supplement: Supplementary file 3 — Supplementary Material 3. [file 13643_2025_3005_MOESM3_ESM.pdf]

Supplementary

Table 1: Patient and tumor characteristics of the training cohort

**Study**	**pN+ (%)**	**Age (years)**	**Tumor size**	**Histological grade (%)**	**Histological type (%)**	**LVI (%)**
Cattell, 2022*	34	pN0: 55.5[a] pN+: 55.1[a]	Missing	pN0: 1 = 25.0, 2 = 51.9, 3 = 23.1 pN+: 1 = 1.8, 2 = 61.8, 3 = 36.4	pN0: NST = 81.5, lobular = 14.8, other = 3.7 pN+: NST = 89.1, lobular = 10.9, other = 0	pN0: 8.3 pN+: 63.6
Chen, 2023	27	51[a]	≤2 cm 46.6 %, > 2cm 53.4%	1 = 8.1, 2 = 50.5, 3 = 41.4	NST = 93.6, lobular = 2.9, other = 3.5	Missing
Cheng, 2022*	47	pN0: 52.0[a] pN+: 52.0[a]	Missing	pN0: 1 = 3.6, 2 = 77.3, 3 = 19.1 pN+: 1 = 0, 2 = 85.7, 3 = 14.3	pN0: NST = 100 pN+: NST = 95.9, other = 4.1	Missing
Choi, 2020	13	pN0: 57[a] pN+: 52[a]	Missing	pN0: 1 – 2 = 47, 3 = 53 pN+: 1 – 2 = 43.9, 3 = 56.1	Missing	pN0: 3.4 pN+: 9.8
Ding, 2020*	34	pN0: 55.6[a] pN+: 55.1[a]	Missing	pN0: 1 = 24.3, 2 = 52.3, 3 = 23.4 pN+: 1 = 1.8, 2 = 61.8, 3 = 36.4	pN0: NST = 81.3, lobular = 15.0, other = 3.7 pN+: NST = 89.1, lobular = 10.9, other = 0	pN0: 8.4 pN+: 63.6
Duan, 2023	41	pN0: 50.5[b] pN+: 50.0[b]	pN0: 18.6 mm[a] pN+: 20.2 mm[a]	Missing	Missing	Missing
Fehr, 2004	42	56[a]	22.3 mm[a]	1 = 17, 2 = 33, 3 = 50	NST = 75, lobular = 21, other = 4	Missing
Gao, 2023	41	pN0: 53.0[a] pN+: 53.4[a]	pN0: 19.3 mm[a] pN+: 25.9 mm[a]	pN0: 1 = 18.1, 2 = 55.1, 3 = 26.8% pN+: 1 = 5.9, 2 = 55.1, 3 = 39	Missing	Missing

Gravina, 2022	17	55[a]	Missing	Missing	Missing	Missing
Jiang, 2021	33	pN0: 54.9[a] pN+: 55.9[a]	pN0: 19.8 mm[a] pN+: 22.3 mm[a]	Missing	pN0: NST = 53, lobular = 39, other = 9 pN+: NST = 48, lobular = 51, other = 2	
Kato, 2019	27	pN0: 61.2[a] pN+: 54.6[a]	pN0: 12.8 mm[a] pN+: 21.2 mm^2	pN0: 1 = 78.1, 2 = 21.9 pN+: 1 = 50, 2 = 50	pN0: NST = 78.1, other 21.9 pN+: NST = 83.3, other = 16.7	pN0: 6.3 pN+: 41.7
Kawaguchi	29	pN0: 58.2[a] pN+: 54.6[a]	pN0: 22.3 mm[a] pN+: 36.2 mm[a]	pN0: 1 = 14, 2 = 85, 3 = 1 pN+: 1 = 0, 2 = 97, 3 = 3	pN0: lobular = 100 pN+: lobular = 100	pN0: 12 pN+: 60
Kim, 2023	21	54[a]	Missing	High = 23.5	NST = 85.7	Missing
Li, 2019*	50	< 40 years = 17.5%, ≥40 years = 82.5%	Missing	Missing	Missing	26.7
Li, 2021*	42	pN0: 51.8[a] pN+: 49.0[a]	pN0: 20.4 mm[a] pN+: 25.4 mm[a]	pN0: 1 = 3.5, 2 = 80.0, 3 = 16.5 pN+: 1 = 1.2, 2 = 81.7, 3 = 17.1	Missing	Missing
Liu, 2019*	34	pN0: 55.5[a] pN+: 55.1[a]	Missing	pN0: 1 = 25.0, 2 = 51.9, 3 = 23.1 pN+: 1 = 1.8, 2 = 61.8, 3 = 36.4	pN0: NST = 81.5, lobular = 14.8, other = 3.7 pN+: NST = 89.1, lobular = 10.9, other = 0	pN0: 8.3 pN+: 63.6
Liu, 2021	38	47[a]	Missing	Missing	Missing	Missing
Ma, 2022	37	50[b]	Missing	1 = 52, 2 = 38, 3 = 10	NST = 58, lobular = 25, other = 17	Missing
Mao, 2020	50	pN0: 48.1[a] pN+: 49.1[a]	pN0: 24.8 mm[a] pN+: 25.8 mm[a]	pN0: 1 = 8, 2 = 46, 3 = 46	Missing	Missing

				pN+: 1 = 10,<br>2 = 40,<br>3 = 50		
Qiu, 2020	32	53.4[a]	Missing	Missing	Missing	Missing
Qiu, 2022	55	pN0:<br>52.9[a]<br>pN+:<br>53.6	Missing	pN0: 1 = 6.3,<br>2 = 43.8,<br>3 = 50.0<br>pN+: 1 = 7.8,<br>2 = 30.8,<br>3 = 61.5	Missing	Missing
Song, 2022	36	pN0:<br>53.8[a]<br>pN+:<br>53.9[a]	pN0: 20.0<br>mm[a]<br>pN+: 20.4<br>mm[a]	pN0: 1 = 29.5,<br>2 = 56.8,<br>3 = 13.7<br>pN+: 1 = 12.3,<br>2 = 60.4,<br>3 = 27.4	Missing	Missing
Song, 2023	20	57.6[a]	Missing	1 – 2 = 72.4,<br>3 = 27.6	NST = 86.8,<br>other = 13.2	10.4
Torstenson,<br>2013	20	All: 65.2[a]<br>pN0:<br>65.6[a]<br>pN+:<br>63.4[a]	Missing	All: 1 = 41.4,<br>2 = 39.2,<br>3 = 19.5<br>pN0: 1 = 42.2,<br>2 = 37.9,<br>3 = 19.9<br>pN+: 1 = 38.0,<br>2 = 44.3 ,<br>3 = 17.7	All: NST =<br>75.8,<br>lobular = 11,<br>other = 13.2<br>pN0: NST =<br>74.8,<br>lobular = 11.8,<br>other = 13.4<br>pN+: NST =<br>79.7,<br>lobular = 7.6,<br>other = 12.6	All: 8.2<br>pN0:<br>4.0<br>pN+:<br>25.3
Wang,<br>2021*	50	pN0:<br>47.9[a]<br>pN+:<br>49.7[a]	pN0: 20<br>mm[b]<br>pN+: 21<br>mm[b]	Missing	Missing	Missing
Wang, 2022	54	55.7[a]	Missing	Missing	Missing	Missing
Yuan, 2023*	41	All: 56.8[a]<br>pN0:<br>57.3[a]<br>pN+:<br>56.1[a]	All: 22.0<br>mm[a]<br>pN0: 19.9<br>mm[a]<br>pN+: 25.0[a]	Missing	Missing	Missing
Zha, 2021	29	55.4[a]	19.0 mm[a]	Missing	NST = 94.3,<br>lobular = 1.3,<br>other = 4.4	16.4
Zhao, 2023	33	56.7[a]	23.7 mm[a]	Missing	NST = 65,<br>other = 35	Missing
Zhou, 2020	50	48.6	≤ 2.0 cm =<br>42%,<br>2.1 – 4.0<br>cm =<br>49.3%,	Missing	NST = 50.7,<br>lobular = 31.5,<br>other = 17.8	Missing

			> 4.0 cm = 77%			
Zhu, 2021	46	All: 46.3[a]<br>pN0: 47.3[a]<br>pN+: 45.1[a]	Missing	All: 1 = 5.7, 2 = 62.6, 3 = 31.7<br>pN0: 1 = 4.5, 2 = 65.7, 3 = 29.8<br>pN+: 1 = 7.1, 2 = 59.0, 3 = 33.9	All: NST = 92.7, other = 7.3<br>pN0: NST = 89.6, other = 10.0<br>pN+: NST = 96.4, other = 3.6	Missing
Zhu, 2022	48	All: 51.9[a]<br>pN0: 51.1[a]<br>pN+: 49.2[a]	Missing	Missing	All: NST = 92.1, lobular = 3.5, other = 4.4	

Abbreviations: lymphovascular invasion (LVI), no special type (NST)

*Complete study cohort
[a]Mean

[b]Median

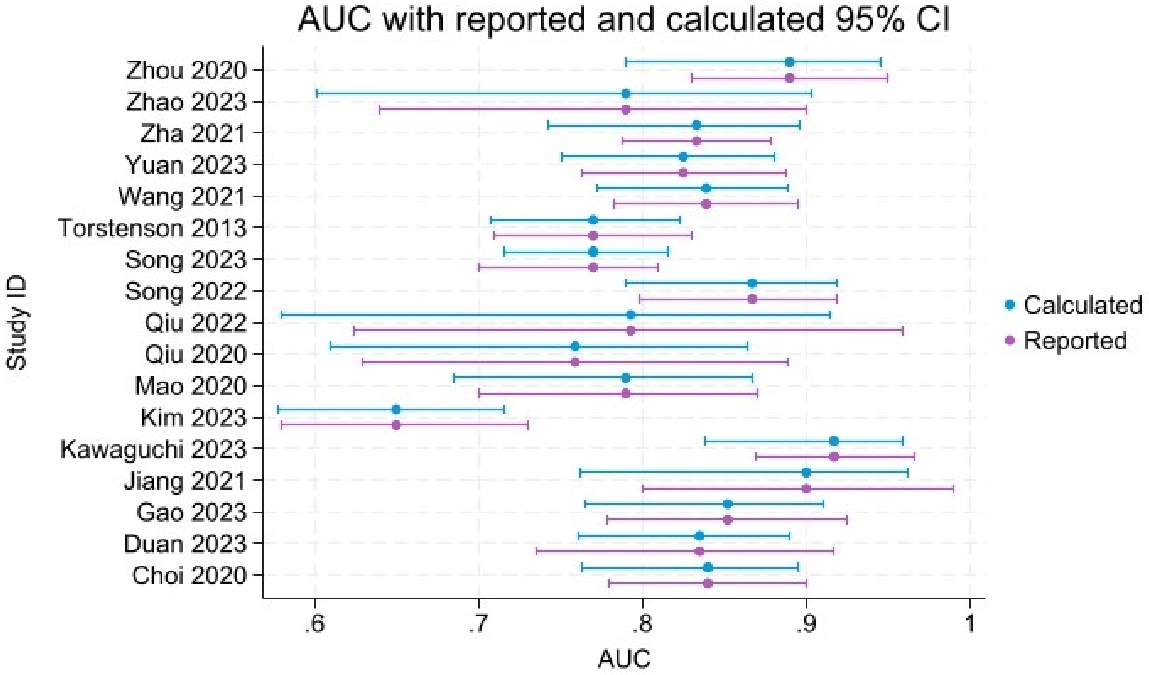

Figure 1: Comparison of reported and calculated confidence intervals for AUCs

Abbreviations: area under the curve (AUC), confidence interval (CI)

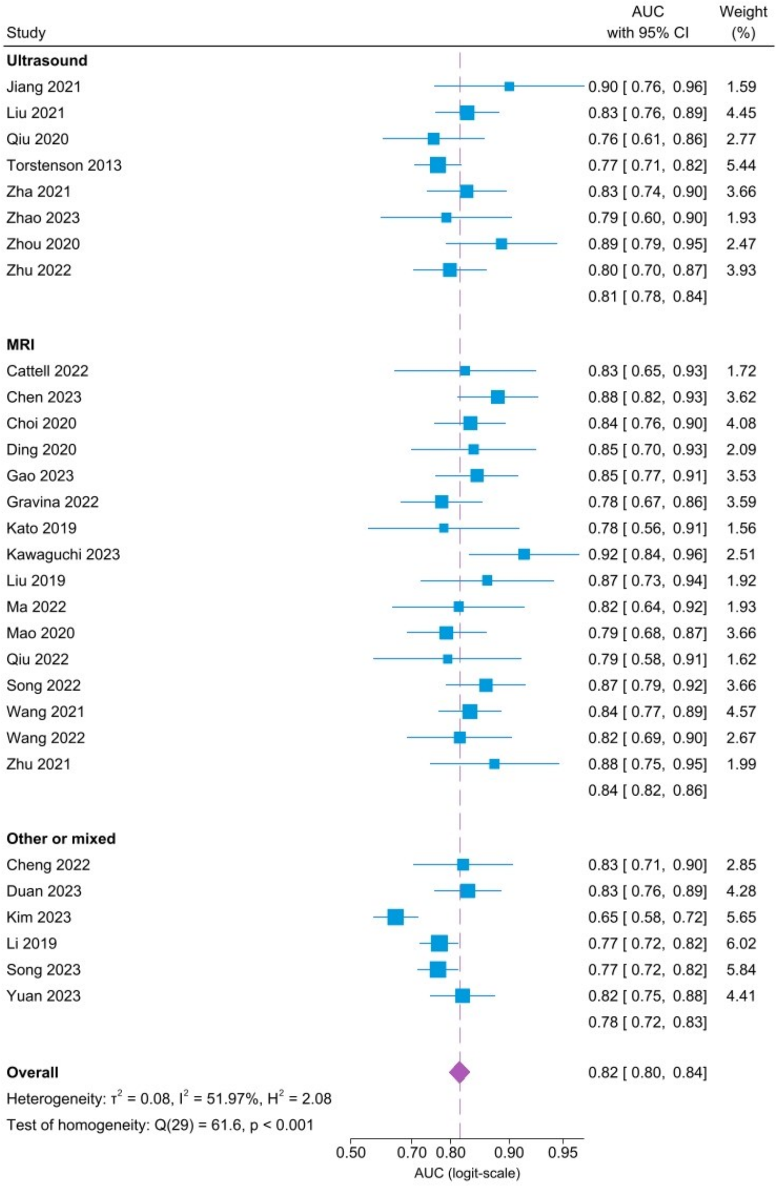

Figure 2: Forest plot of overall performance and heterogeneity including all studies (n = 30)

Abbreviations: area under the curve (AUC), confidence interval (CI)
